# Predispensing of Antivirals to High-Risk Individuals in an Influenza Pandemic

**DOI:** 10.1371/currents.RRN1007

**Published:** 2009-11-12

**Authors:** Edward Goldstein, Joel C. Miller, Justin O'Hagan, Marc Lipsitch

**Affiliations:** Harvard School of Public Health

## Abstract

We consider the net benefits of predispensing antivirals to high-risk individuals during an influenza pandemic, where the measure of the benefit is the number of severe outcomes (such as deaths or hospitalizations) prevented by antivirals in the whole population. One potential benefit of predispensing is that individuals to whom antivirals have been predispensed may be able to initiate treatment earlier than if they had to wait to obtain and fill a prescription, reducing their risk of progression to severe disease. If this benefit exceeds the side effects of misuse for the category of individuals to whom antivirals were predispensed, and if antiviral supply exceeds overall population demand (which appears relevant for several countries including US in the current H1N1 pandemic), predispensing a quantity of antivirals not exceeding the difference between supply and demand is always beneficial. In this paper we consider the net benefits of predispensing antivirals under various scenarios, including demand exceeding supply, and derive mathematical conditions under which antiviral predispensing is advantageous on balance. For individuals whose relative risk of severe outcome is high enough, such as immunosuppressed individuals (particularly children) and possibly individuals with neurological disorders, predispensing is always beneficial at a given level of antiviral stockpile with modest assumptions on the relative benefit of early treatment by a predispensed course, regardless of the overall population demand for antivirals during the course of an epidemic. Making additional assumptions on either the overall population demand for antivirals (which appear relevant in the current situation) or on the relative benefit of predispensing would make predispensing net beneficial with inclusion of a larger number of persons such as pregnant women and morbidly obese adults.

A key objective for the response to the autumn wave of pandemic influenza A/H1N1 (H1N1pdm) in the Northern Hemisphere is to reduce severe morbidity and mortality that would result from an unmitigated pandemic.  Broadly, such responses may be divided into two groups: (a) efforts to reduce population-wide transmission of the virus and thereby protect individuals from becoming infected, either permanently (vaccination) or for a period of time until vaccines become widely available (non-pharmaceutical interventions), and (b) efforts to protect individuals at particularly high risk of complications from becoming infected or, if infected, from developing severe disease.  Here we concentrate on aspect (b) of the response via the usage of antiviral drugs.

The ability of antiviral drugs to alleviate symptoms and shorten their duration is well documented for seasonal influenza, particularly if antivirals are taken during the earlier stages of influenza infection [1] [2] [3]. A 2003 study suggests a 60% reduction in influenza hospitalization rates for patients who received early antiviral treatment for seasonal influenza [4]. For H1N1pdm, recent data [5] show that among hospitalized patients, severe outcomes (ICU admission and/or death) were less likely in patients who received antiviral treatment within two days of symptom onset; the same conclusion is true for lethal outcomes in hospitalized patients in [6]. 

Several recent studies suggest that most severe outcomes for H1N1pdm infection occur in individuals with pre-existing medical conditions [5] [6] [7] [8] [9] [10] [11] [12], though the exact percentages vary between the studies.  This has been recognized in several countries, where prompt antiviral prescription to symptomatic high-risk individuals is recommended [13] [14] [15] [16] [17]. However in several countries, including US, such a prescription requires a doctor visit. Such a visit may be problematic to symptomatic individuals with underlying health conditions and may not occur promptly enough for various reasons, including a possible wait for symptoms to go away. Predispensing antiviral drugs so that individuals in high-risk groups have access to medications immediately upon becoming symptomatic without the need to seek a prescription or fill it in a time of possible scarcity may be a valuable strategy to reduce their risk of severe disease or mortality.  Predispensing here is defined as a policy (most likely implemented by an individual physician) of prescribing and urging a patient to fill the prescription for a course of neuraminidase inhibitor in advance of any known infection with H1N1pdm.  The patient would be instructed to begin self-treatment (possibly following communication with the physician) upon meeting a definition of a suspected H1N1pdm infection. 

The benefit of using a predispensed course of antivirals vs. one obtained by prescription is hard to assess.  However if one believes that such a benefit does exist and exceeds the side effects for the category of individuals to whom antivirals were predispensed, predispensing of antivirals is always advisable in a situation when the population demand for antivirals is smaller than the available supply.  This is because predispensing a quantity of antivirals not surpassing the difference between supply and demand would not deprive anybody of an antiviral course, and a predispensed course is more likely to prevent a severe outcome than a course obtained by prescription during the epidemic. The scenario of supply exceeding demand appears relevant to several developed countries with a large supply of antivirals, and evidently to the US due to strict current prescription guidelines for low risk individuals [13].  This simple rationale is perhaps the main practical motivation behind antiviral predispensing in a current H1N1 epidemic.

In a scenario when demand exceeds supply, there is additional benefit to predispensing having to do with the fact that a predispensed course of antivirals would end up in the hands of a high-risk rather than a low-risk person, and thus is more likely to prevent a severe outcome.  There is also harm to predispensing in this case resulting from the fact that some antivirals will be in the hands of individuals who will not need them, and hence will be wasted, depriving others who do need them of access.

In this paper we examine the net benefits of predispensing antivirals under various levels of  population demand.  We define conditions under which predispensing a defined quantity of antivirals, one course each to a subpopulation at high risk of death or hospitalization from H1N1pdm infection would provide a net benefit in terms of reducing severe outcomes compared to a policy of leaving them in state and national stockpiles for distribution only to infected patients. At a given level of antiviral supply and relative benefit of early treatment there may be some groups of individuals with sufficiently high relative risk of dying such that predispensing is always beneficial regardless of the overall population demand for antivirals during the course of an epidemic.  Under certain assumptions about the overall demand for antivirals  -- namely, that it is not too close to the total supply – one can show that there is a net benefit to predispensing to a larger class of high-risk persons (those with risk above the population average, but not in the highest-risk category). We will attempt to quantify these situations in the context of limited available data on severe outcomes in high-risk individuals.

## **Relative risks for certain high-risk groups**

In this section we attempt to assess relative risks for a severe outcome from H1N1 infection for certain high-risk groups for which data is available. Our data sources are [5] [6] [8] [18] [19]. The estimates below are crude and based on limited available data. Also throughout this section, a relative risk for a severe outcome for a high-risk group is the ratio of the rate of this severe outcome (per capita) in this high-risk group over the rate of that severe outcome in the whole population. 

Pregnant women constitute 1% of the US population [8]. An estimate in [8] suggests that they are about 4 times more likely to be hospitalized due to H1N1 infection compared to general public. The percentages of pregnant women among hospitalized patients in [5] and [6] are 6.6% and 9.6% correspondingly.  The percentage of pregnant women among deaths in [6] is 5.1%; the percentage of pregnant women among deaths or ICU admissions in [5] is 9%. We therefore estimate the relative risk for hospitalization for pregnant women to be between 4-9.6, and the relative risk for death or ICU admission to be between 5.1-9.

Morbidly obese adults (BMI $\geq 40$) constitute 4.8% of the adult US population [19], and correspondingly 3.63% of the of the whole US population. Data in [6] suggest that morbidly obese adults constitute 18.6% of hospitalized cases, and 31.5% of fatal cases.  Data in [5] (which has a lower percentage of adults compared to [6]) suggests that 14.3% of hospitalized patients were morbidly obese adults; among patients who died or were transferred to ICU, 20.6% were morbidly obese adults. Thus we estimate that the relative risk for hospitalization for morbidly obese adults ranges in 3.95 – 5.11, while the relative risk for ICU admission or death ranges in 5.68 – 8.68. 

We draw our data on the percentages of immunosuppressed individuals in different age groups in the US from [18] (kindly provided by Diane Wagener). The National Health Interview Survey (NHIS), conducted annually by the Centers for Disease Control and Prevention (CDC), was used as a principal source for prevalence data in [18]. Specific sources are: Cancer in past 3 years 2006 data (National Health Interview Survey, 2006); HIV/AIDS 2006 data CDC Surveillance, 2007 Report http://www.cdc.gov/hiv/topics/surveillance/resources/reports/2007report/default.htm; ESRD (US Renal Data System, Table D.11); Transplants data (Organ procurement and Transplantation Network, 2009).

Immunosuppression is very rare in children, accounting for less than 0.18% of the population of children, thus less than 0.044% in the overall population [18]. At the same time, immunocompromised children represent 6.4% of all H1N1-related hospitalizations in [5] and 5.3% of all H1N1-related hospitalizations in [6]. They also represent 3/118 (2.5%) of all deaths in [6]. Thus we estimate the relative risk for hospitalization to be between 120-145, and the relative risk for death to be 57. Another way to look at those numbers is to note that there are around 130,000 immunocompromised children in the US. There were 17838 laboratory-confirmed influenza associated hospitalizations in the US between August 30 and October 31 [20]. Assuming a total of 30000 H1N1pdm-associated influenza hospitalizations in the US and the percentages of immunocompromised children among the hospitalized from [6] [5], we get that roughly 1 in 75 immunocompromised children is hospitalized with H1N1 in the US. 

Immunocompromised adults represent 1.9% of the US population [18]. They represent 10.2% of all hospitalizations and 30.5% of all fatalities in [6]. Immunocompromised adults represent 10.7% of hospitalizations in [5]. Immunocompromised individuals represent 17.9% of all deaths or ICU admissions in [5]; presumably most of them are adults. Thus we estimate the relative risk for hospitalization for immunocompromised adults to be between 5.4 – 5.6, and their relative risk for death between 9.4 – 16.1.

We have no data on prevalence of individuals with neurological (neurocognitive and neuromuscular) disorders.  At the same time, children with neurological disorders represent 9.9% of all hospitalizations in [5]. Children with neuromuscular disorders represent 4.5% of all hospitalizations, and 3.4% of all deaths in [6]. Moreover children with neurological disorders have an increased relative risk for respiratory failure given hospitalization [21]. Adults with neuromuscular disorders represent 11.9% of all deaths in [6].  People with neurocognitive or neuromuscular disorders each represent 13.4% of deaths or ICU admissions in [5].

## **Benefits and harms of predispensing**

To fix notation, we assume here that we seek to optimize the number of lives saved (i.e. minimize the number of lives lost due to H1N1pdm); however, with suitable modifications to the definitions of all terms, we could equally attempt to prevent hospitalizations or intensive care admissions.   There are at least two possible benefits, and three harms, to predispensing.  The first benefit of predispensing is that a patient possessing a predispensed course of antivirals will likely begin therapy earlier in the course of H1N1pdm infection than one must acquire a prescription (possibly including a visit to a physician) and fill it, thereby most likely delaying treatment.  This benefit accrues whether or not there is a shortage of antivirals, since it simply reflects the time required to acquire and fill the prescription.  A second possible benefit occurs if the demand for antivirals exceeds supply, so that not all patients who attempt to acquire antivirals can do so.  In this situation, predispensing a course assures that it is in the hands of someone who, if untreated, would be likely to develop severe disease, rather than (potentially) going to someone who will not likely develop severe disease, and whose benefit from taking the antiviral would therefore be less. 

The first harm of predispensing occurs only if total demand for antivirals exceeds supply, namely, a predispensed course is unavailable to anyone who may need it other than the person who has received it.  If demand exceeds supply, this means one more person, possibly someone who would benefit greatly from having the antiviral is unable to obtain it. A second harm of predispensing is the possibility that a predispensed course will be used by a high-risk individual who would not otherwise be able to obtain an antiviral drug, and this usage will lead to a severe side effect.  We have no way of assessing the likelihood of such an outcome; in particular, the major severe side effects reported to date are neuropsychiatric events in oseltamivir recipients, and the manufacturer’s data review concludes that there is no evidence of a causal link between oseltamivir and these adverse events [22].  We will not model this second harm in our analysis. Yet another possible harm to predispensing is preliminary evidence that people treated with antivirals have lower levels of antibodies compared to people who weren’t. We are not aware of data quantifying additional risk for re-infection, if any, after antiviral treatment compared to no antiviral treatment. Therefore we will not model this potential harm either.

To assess the net benefit or harm from predispensing, we define some notation, summarized in Table 1.  We divide the total population into two groups: a *high-risk group* for which we are considering predispensing, and *the general population*, which is comprised of all individuals not in the high-risk group.  These constitute respectively a proportion \begin{equation*}q\end{equation*}  (high risk) and  \begin{equation*}1-q\end{equation*}  (general population) of the total population.  Within the general population, risks may vary, so some individuals may be at higher risk than others.   To keep the exposition simple we assume throughout that under predispensing, all \begin{equation*}q\end{equation*} members of the high-risk group receive the predispensed drug; the sufficient conditions for predispensing to be beneficial still hold if uptake is less than perfect, as it will be in practice.  All quantities in our notation are defined as proportion of the total population, hence lie between 0 and 1.  Let *T* be the total supply of antivirals (as a proportion of the population); thus, if enough antivirals are available for treatment of 20% of the population, then *T*=0.2 .  Let *D* be the demand for antivirals in the general population, that is the number of individuals not at high risk who would receive antiviral treatment in the general population if supply were not limited; in practice, some of this demand may be unmet as the supply is constrained.  Let \begin{equation*}p_d\end{equation*} be the probability of dying from the infection over the whole course of the epidemic in the whole population (if no antivirals are used); the death probability in a high risk group is \begin{equation*}Rp_d\end{equation*} .  Note that these death probabilities are not conditional on infection (i.e., are not case-fatality proportions) but are unconditional, reflecting the risk of infection times the risk of dying from infection.  Here the number \begin{equation*}R\end{equation*}  is the relative risk of dying in a high risk group compared to the general population.  \begin{equation*}R\end{equation*} can be estimated from the existing epidemic data while \begin{equation*}p_d\end{equation*} may be hard to estimate a priori; as we shall see, \begin{equation*}p_d\end{equation*} is factored out of our equations and need not be known. Let \begin{equation*}p_S\end{equation*}  be the probability that a course of antivirals obtained from a stockpile during the epidemic would save a life of a person who would die otherwise.  We assume that this probability is the same between high-risk individuals and members of the general population.  This key assumption may be incorrect, and is discussed later.  Let \begin{equation*}Bp_s\end{equation*} be the probability that a predispensed course of antivirals would save a life of a high risk person who would die otherwise.  \begin{equation*}B\end{equation*}  can be thought of as  the “relative benefit” in preventing mortality of receiving a predispensed course of antivirals for a high risk person who takes it, compared to receiving a non-predispensed course. \begin{equation*}B\end{equation*}  captures the benefit of early vs. delayed treatment; however, \begin{equation*}B\end{equation*}  also may be reduced to the extent that a predispensed course is taken for non-influenza illness, in which case it cannot save a life.  Thus \begin{equation*}B\end{equation*}  exceeds one to the extent that early treatment is better than delayed treatment, but it is decremented in proportion to the probability that the course is wasted before it is needed. 

Let  \begin{equation*}L\end{equation*}  be the total number of people in the general population who would die during the epidemic and whose lives would be saved if they received antivirals upon demand during the course of the epidemic.  Since the total number of people in the total  population who would die during the epidemic and whose lives would be saved if they received antivirals during the course of the epidemic is \begin{equation*}p_dp_s\end{equation*} , clearly \begin{equation*}L\leq p_dp_s\end{equation*} .  We make additional assumptions about how the probability of receiving antivirals behaves if demand exceeds supply; these are made explicit in Appendix A. 

TABLE 1: Parameters of the model, as a proportion of the total population (including high-risk and general populations)

**Table:** 

**Parameter **	**Definition**
\begin{equation*}T\end{equation*}	Supply of antivirals, before predispensing, as a fraction of total population size
\begin{equation*}D,D'\end{equation*}	Number of antiviral courses used by the general population ( \begin{equation*}D\end{equation*} ) or total population ( \begin{equation*}D'\end{equation*} ) if no supply constraint, as fraction of the total population size
\begin{equation*}L\end{equation*}	Total number of lives in the general population that would be saved under no predispensing, if no supply constraint
\begin{equation*}q\end{equation*}	Proportion of persons in the high risk group to whom predispensing of one course is considered, hence also the number of courses considered for predispensing, as a fraction of the total population
\begin{equation*}p_s\end{equation*}	Probability that a course of antivirals obtained during the epidemic would save a life of a person who would die otherwise, absent any predispensing
\begin{equation*}B\end{equation*}	Relative benefit (in terms of probability of saving life) for a high risk person to be treated early (with a predispensed course) compared to being treated with a non-predispensed course. Thus \begin{equation*}p_SB\end{equation*} is the probability that a predispensed course of antivirals would save a life of a high risk person who would die otherwise
\begin{equation*}p_d\end{equation*}	Death rate from the epidemic in the whole population, if no antivirals were used
\begin{equation*}R\end{equation*}	Ratio between the death rate in the high risk group and the general population.

With this notation in place, we can define the conditions under which it is advantageous to predispense 1 course each to a proportion \begin{equation*}q\end{equation*}  of the population.  Our main result is the following: 

MAIN RESULT: Predispensing saves more lives than not predispensing when any of the following conditions hold:

(i) Supply of antivirals exceeds demand even after predispensing, and \begin{equation*}B>1\end{equation*} . 

 (ii) Demand \begin{equation*}D\end{equation*}  in the general population exceeds supply even without predispensing, and the following conditions are met:      

\begin{equation*}B> \frac{T}{D}\end{equation*}  and  \begin{equation*}R>\frac{1}{D(B-T/D)}\end{equation*}

 (iii)         Supply exceeds demand without predispensing, but after predispensing, demand exceeds supply,$(T-q\leq D\leq T)$ and the following conditions are met:

\begin{equation*}B> \frac{T}{D}\end{equation*}  and    \begin{equation*}R>\frac{D+q-T}{Dq(B-1)}\end{equation*}. 

(iv)    The above results show that even if demand cannot be predicted, predispensing is advantageous when

\begin{equation*}B>1\end{equation*}  and      \begin{equation*}R>\frac{1}{T(B-1)}\end{equation*}

The justification of this result and the assumptions underlying it are presented in Appendix B.  Condition (iv) tells us when a group is at high enough risk that it is worth predispensing to them even if we have no idea of the expected antiviral demand.

This result can be seen graphically in Figures 1-2, which considers a hypothetical case in which there is a supply adequate for \begin{equation*}T=0.2\end{equation*}  of the population, and predispensing to \begin{equation*}q=3\%\end{equation*}  of the total population is under consideration.  The parameter that varies between the figures is the relative benefit $B$ of saving a life by a predispensed course vs. a course obtained by prescription. We are not aware of data allowing an assessment of this parameter; we consider 2 scenarios: $B=2$ (Figure 1), $B=1.3$ (Figure 2). 

 The far left side of each of the figures shows low levels of demand, in which there is no harm to predispensing, so predispensing to any group for which $B>1$ may be beneficial.  At the far right, competition for antivirals is strong, so all antivirals are used, mostly by the general population, and it is beneficial to predispense even to a group that gets modest benefit from antivirals in order to capture the benefit of early treatment.  In the middle, when demand is similar to supply, it is beneficial to predispense only to groups that benefit disproportionately from antivirals ($R>5$ for  $B=2$,  $R>16.7$ for $B=1.3$). 

**Figure:**
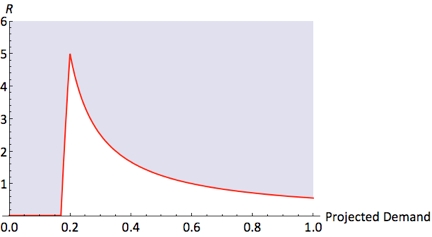
FIGURE 1: Predispensing is beneficial (shaded area) when \begin{equation*}R\end{equation*}  exceeds a certain threshold that depends on the projected demand.  Here a supply adequate for \begin{equation*}T=20\%\end{equation*}  of the population is assumed, and predispensing to \begin{equation*}q=3\%\end{equation*}  of the total population is under consideration.  Also, we assume that treatment of a high-risk person with a predispensed course is twice as likely to be life-saving as if treatment is with a non-predispensed course, because predispensing allows earlier initiation of treatment ( \begin{equation*}B=2\end{equation*} ).  For low projected demand (below \begin{equation*}D<T-q=0.17\end{equation*} ), there is no harm to predispensing, and predispensing even to groups that will gain below-average benefit from antivirals is better than no predispensing.  For very high projected demand, ( \begin{equation*}D\end{equation*}  near 1), predispensing is beneficial even for very modest values of $R$, because competition for antivirals is strong, and predispensing has a relative benefit compared to acquisition by ill persons.  For projected demand comparable to the supply ( \begin{equation*}D\approx T=0.2\end{equation*} ), predispensing is valuable only for groups for whom antivirals are considerably more valuable than the general population, because each course predispensed comes at the expense of someone else who could use it, yet it does not strongly increase the chance that a high-risk individual gets treated (since that individual would likely receive a course anyway without predispensing). 

**Figure:**
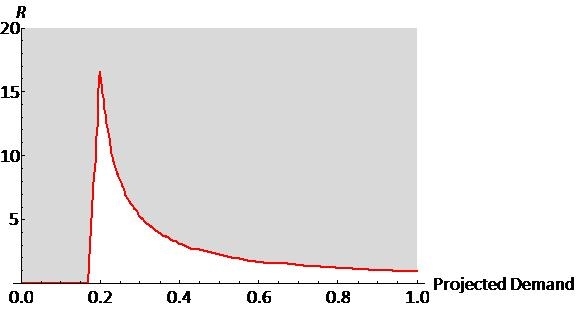
*FIGURE 2: If predispensed courses are 1.3 more likely to be lifesaving than non-predispensed courses taken by the same person, the conditions for benefits of predispensing are more restrictive.  Parameters are as in figure 1, except that $B=1.3$   In particular, in this situation if demand=supply, it is only beneficial to predispense a course if $R>16.7$*

Finally, we note that while it is beneficial to predispense antivirals if either of the conditions in the Main Result are met, it is not true in general that the benefit increases with the quantity of antivirals predispensed. Under certain conditions it would be better to predispense to a subset of the high-risk population.  This is discussed further in Appendix C, where a simple criterion is given ensuring that each successive predispensed dose increases the net benefit, provided that the quantity of antivirals predispensed is not too large.

## **Interpretation of these Conditions**

Predispensing is most advantageous for groups that have the highest mortality rate (high \begin{equation*}R\end{equation*} ) and that benefit most from predispensed antivirals compared to non-predispensed ones (high \begin{equation*}B\end{equation*} ).  These factors capture the demand-independent aspect that it is useful to position antivirals with individuals who can be most helped by having them close at hand, rather than getting them after they become ill.  A second benefit of predispensing is to ensure access of antivirals to those who will benefit the most from them. This benefit is greatest when demand is very high, because in such circumstances the high-risk individuals are very likely to need antivirals, but not to get them.  Thus the benefits of predispensing are greatest for larger values of \begin{equation*}R\end{equation*} , \begin{equation*}B\end{equation*} , and \begin{equation*}D/T\end{equation*} .  The costs of predispensing – lost opportunities for individuals who do not receive predispensed courses – are zero when demand is low enough (as all who need the antivirals can receive them) then increase, but not as fast as the benefits.  Qualitatively, then, predispensing is most advantageous when demand is very high or very low, and least likely to be advantageous when demand just exceeds supply.  Even there, however, if the benefit of a predispensed course is significantly larger than that of a non-predispensed course, predispensing is valuable for individuals who can benefit more from treatment than the general population. 

## **Risks of Predispensing**

The major risk of predispensing is that courses that would otherwise be life-saving (when used from the stockpile) will be predispensed, then not used by someone for whom they would be more likely to be life-saving.  We can consider what has been written so far from another perspective: this risk is most acute when demand is near supply ( \begin{equation*}T\approx D\end{equation*} ) , when individuals not in the group to be predispensed would benefit from treatment by predispensed antivirals which won’t be used, and when predispensing and consequent early use are not very beneficial (reducing \begin{equation*}B\end{equation*} ). 

The idea of predispensing was previously considered in the context of pre-pandemic sale of antiviral “Medkits,” special packaging of oseltamivir or zanamivir for home storage to be saved until a pandemic occurred.  A number of concerns were raised regarding such Medkits, resulting eventually in a lack of approval by the Food and Drug Administration (www.fda.gov/ohrms/dockets/ac/08/minutes/2008-4385m1-final.pdf). One of these concerns was the issue of equity - if Medkits were to be purchased by individuals, individual decision making and ability to pay would influence access. Another concern was the risk of inappropriate use or wastage.  A key difference between pre-pandemic distribution of antivirals and predispensing just prior to or in the middle of a wave of pandemic influenza is that the time frame for wastage is much shorter in the present case. Thus, we expect that $B$ will primarily reflect the advantage of early vs. delayed treatment, for which there is evidence [1] [2] [3] [4] [5]. 

It is also worth mentioning that unlike the situation with antibacterial medications – which can promote drug resistance even when used to treat non-bacterial infections, because of their effect on bystander flora [23], anti-influenza drugs do not promote resistance when used to treat non-influenza infections.  In the current H1N1 pandemic, resistance levels appear to be very low – for instance in the UK, where extensive antiviral usage takes place, resistance levels were estimated to be 0.17% [24]. Moreover, prior mathematical modeling studies [25] [26] have shown that if the epidemic is already large (as it is now) at the time that large-scale antiviral treatment begins, then resistant strains will be unlikely to ascend to high frequency in the population before the epidemic is over, even in the absence of vaccination. Thus we think that additional antiviral usage which may result from predispensing will have a very minor impact on antiviral resistance levels in the current H1N1 pandemic.****

## **Benefits of early antiviral treatment for preventing severe outcomes.**

A key source of uncertainty in our estimates is the relative benefit $B$ for preventing a severe outcome by a predispensed course of antivirals vs. one obtained by prescription by an individual who would die otherwise. In this section we describe data from [5] [6] showing a reduction in probability of a severe outcome resulting from early antiviral treatment. We also explain why it is difficult to estimate $B$ from data. 

Data in [6] show that among hospitalized H1N1pdm patients, 18/357 (5%) of those who received antiviral treatment within 48 hours died; the same numbers for all patients who received any antiviral treatment are 70/701 (10%).  If we think of those hospitalized patients who received antivirals within 48 hours as a proxy for the predispensed individuals who would progress into a severe condition, and all individuals in the study who received antivirals as a proxy for individuals who would progress into a severe condition and receive antivirals during the epidemic, then ratio of probabilities 1.98=(70/701)/(18/357) can be though of as a ratio of probabilities of dying given that you receive antivirals upon infection during the course of the epidemic vs. having a predispensed course.   

Data in [5] show that among hospitalized H1N1pdm patients, 13/75 (17.3%) of those who received antiviral treatment within 48 hours were either transferred to ICU or died; the same numbers for all patients who received any antiviral treatment are 56/200 (28%).  Using the same proxies as above, the ratio of probabilities 1.62=(56/200)/(13/75) can be though of as a ratio of probabilities of being transferred to ICU or dying given that you receive antivirals upon infection during the course of the epidemic vs. having a predispensed course.

Those proxies may be inaccurate. Having antivirals predispensed may work better than merely receiving them within 48 hours after symptom onset. Additionally, both datasets are based on a time period before the CDC guidelines on Sep. 8, 2009 [13] recommending prompt treatment of symptomatic high-risk individuals without a lab test confirmation; at the same time, those datasets address hospital patients, many of whom received antivirals promptly, presumably upon a first doctor visit.

Regardless of whether the proxies are accurate, they do not yield enough information to get an estimate of $B$, the relative benefit of preventing a severe outcome (death or ICU admission) by a predispensed course vs. a course obtained upon infection by a person who would progress into this outcome otherwise. To see that, coming back to the study in [5], let $p$ be the proportion of hospitalized patients who would die or need to be transferred to ICU unless they receive antivirals. The relative benefit of preventing a severe outcome is then 

         
                                                       

$\frac{p-0.173}{p-0.28}$

Here the numerator represents individuals who were spared a bad outcome because of early antiviral treatment, and the denominator represents individuals who were spared a bad outcome because of any antiviral treatment. Thus if $p=0.35$, $B=2.52$; if $p=0.5$, $B=1.48$ etc. Data in [5] show that among hospital admissions who never took antivirals, 9/68 (13.25%) either died or were transferred to ICU. It appears that people who did not take antivirals fared best; for the data in [6], they fared slightly worse than people who took antivirals after 48 hours since symptom onset. This is due to a bias that we already encountered in [6] – bad, deteriorating cases were given late antivirals; as a consequence, the group without antivirals has a disproportionate share of good cases. In other words, given high levels of treatment, the reverse causal relation between treatment and outcome makes an estimate of the treatment effect difficult. 

While we cannot render an estimate of $B$ from data, we note that 59/268 (22%) of patients in [5] neither received antivirals nor died or were admitted to ICU. Thus we estimate that $p\leq 1-0.22=0.78$ and hence $B\geq 1.21$. An equality would be a very conservative estimate, which assumes that all the 144 individuals who were treated with antivirals and did not die and were not transferred to ICU had a good outcome because of antiviral treatment. There are additional reasons why this is an underestimate of $B$. Data from [27] suggests that 17% of H1N1 deaths were never hospitalized, and some of them could have benefited from predispensing. Additionally our estimates on the chances of avoiding ICU admission or death are based on hospitalized patients, while some people could avoid hospitalization by using a predispensed course of antivirals.  We believe that in reality $B$ should be bigger than 1.21. 

## **Implications for Decision Making**

On current evidence, we believe that there are groups in the population for whom the relative risk $R$ of death or of other severe outcomes, such as hospitalization, substantially exceeds 1.  These groups include pregnant women [5] [8] [6] [28], children and adults with neurological conditions [5] [6] [29], persons with immunosuppression and certain chronic diseases [5] [6] [13],  morbidly obese individuals (BMI $\geq 40$) [5] [6], and some of the other high-risk groups  [5] [22] [13].  We believe it is also plausible to expect that predispensed antivirals are more likely to be life-saving (or to prevent hospitalizations or other severe outcomes) than those that are not predispensed ($B>1$), because wastage is relatively unlikely given the short time frame between when predispensing and usage could occur, and because early treatment is likely to have benefits in preventing progression to more severe condition [13] [5] [6].  

At the same time, given the generally mild nature of H1N1 infections, a number of developed countries with large antiviral stockpiles will likely have antiviral supply greatly exceeding population demand. The same conclusions appear to be valid for the US, which has a relatively small stockpile compared to some countries, but also a restrictive antiviral usage policy for low-risk individuals [13].  Under these conditions**, **predispensing a quantity of antivirals not exceeding the difference between supply and demand is always beneficial.

Even if one is uncertain about the population demand for antivirals during the course of the epidemic, one can include certain categories of individuals for predispensing given a measure of belief about the relative benefit $B$.  We concentrate on the situation in the US; in particular, we assume that the available antiviral stockpile can cover 20% of the population. 

If one thinks that $B$ is as low as 1.3 (Figure 2), one can still, regardless of the demand levels, include certain groups like immunocompromised children. One should probably also include immunocompromised adults due to a high relative risk for fatality (16.1) resulting from a large study [6]. People with neurological disorders should probably also be included due to high rates of hospitalizations, fatalities and ICU admissions in [5] [6]. If one thinks that $B$ is at least 2 (Figure 1), one should include additional groups in the predispensing category, such as pregnant women, morbidly obese adults, and possibly some additional groups for which we have no prevalence data. 

We again want to emphasize that the above considerations apply given uncertainty whether supply surpasses demand. If one believes that supply does exceed demand (which appears to be relevant for several countries, including US), predispensing a quantity of antivirals not exceeding the difference between supply and demand is beneficial.

## **Discussion**

In the setting of an autumn wave of pandemic influenza in developed countries of the Northern Hemisphere that already possess significant antiviral stockpiles, predispensing of a portion of these stockpiles to individuals at high risk of severe outcome of infection may be a means to prevent death and other severe outcomes by improving the efficiency of use of a limited stockpile.  In this paper we consider the net benefits of a predispensing policy, where the measure of the benefit is the number of severe outcomes (such as deaths or hospitalizations) prevented by antivirals in the whole population. 

Determination whether such a policy is beneficial depends on the assumptions one makes. One general set of assumptions which appears relevant to a number of developed countries is as follows: antiviral supply exceeds demand, and predispensing antivirals is beneficial to the category of individuals to whom antivirals were predispensed. Under those assumptions, predispensing a quantity of antivirals not exceeding the difference between supply and demand is always beneficial. 

If one is uncertain about the overall population demand for antivirals, predispensing to certain high-risk groups may still be justified under the assumptions of our Main Result and Appendix A; our conclusions based on available H1N1pdm data are summarized in Implications for Decision Making section. One additional assumption that should be highlighted in this context is that, as used in the absence of predispensing, antivirals are equally likely to save the life of a treated member of the high-risk group as a treated member of the general population given that both of them would die without treatment.  If treatment is less effective among members of high-risk groups who would die without treatment, then the benefits of predispensing are reduced; if it is more effective, they may be increased.

A key source of uncertainty in our estimates is the relative benefit $B$ for preventing a severe outcome by a predispensed course of antivirals vs. one obtained by prescription by an individual who would otherwise have a severe outcome. This benefit should be assessed against a strategy of identifying high risk persons, educating them to contact their provider for symptoms, and encouraging providers to provide rapid empiric therapy including via telephone. While there is no data to assess $B$ directly, we have estimated the reduction in probability of a severe outcome by early antiviral treatment based on data in [5] [6]. [4] gives additional evidence that early antiviral administration is beneficial in preventing severe outcomes. Similarly, there is evidence that a policy allowing for broader vs. more limited use of antivirals in symptomatic individuals is beneficial for reducing mortality [30]; this benefit was observed both on a population level (Chile vs. Argentina), and in a category of pregnant women. 

We have avoided cost-effectiveness considerations in our analysis as there are several unknowns involved, including the cost of predispensed antivirals (government vs. private rates). We note however that for some groups with the highest risk for severe outcomes, such as immunocompromised children, predispensing may be, among other things, cost effective.

Yet another potential issue related to predispensing is complacency, namely individuals with predispensed antivirals might get a sense of false security  and not exercise enough care in terms of avoiding getting infected. On the other hand, people with high risk conditions to whom drugs were predispensed when they are not sick may be more alert about the risks that influenza poses to them; predispensing may also heighten awareness about high risk groups in the medical community.

Finally, we would recommend that the total proportion of the stockpile predispensed be limited (perhaps to 20% of the stockpile or less). There are several reasons for that. One of them is that the conditions defined in this paper guarantee that predispensing is beneficial in terms of decreasing the mortality provided certain assumptions about temporal patterns of antiviral distribution hold. The most flexible assumption we require is assumption b) in Appendix A. This assumption may be violated under the following scenario: antiviral supply nears depletion and only the most severe cases get antivirals. To deal with such a scenario, we recommend to set aside a certain quantity of antivirals for safekeeping.

## **Acknowledgments**

We thank Richard Hatchett for helpful discussions.  

The MIDAS High Risk Segmentation Group (D. Wagener, R. Zimmerman, D. Lauderdale) kindly shared the data from [18].

## **Funding Information**:

This work was funded by the US National Institutes of Health Models of Infectious Disease Agent Study Cooperative Agreements 5U01GM076497 and 1U54GM088588 to ML for the Harvard Center for Communicable Disease Dynamics (ML,EG,JO), and by the RAPIDD program of the Science & Technology Directorate, Department of Homeland Security, and the Fogarty International Center, National Institutes of Health (JCM). 

## **Competing Interests**

ML discloses consulting fees from the Avian/Pandemic Flu Registry (Outcome Sciences), funded in part by Roche.** ** All other authors declare no competing interests.

****

## **APPENDIX:**

****

### **A.  Justification of the Main Result**

In this Appendix we derive the main result by calculating or finding a bound on the number of lives saved by antiviral treatment in the high risk group (a proportion \begin{equation*}q\end{equation*} ) and in what we term the “general population,” which is the total population without the high risk individuals, a proportion  \begin{equation*}1-q\end{equation*} .  

Let us first list the assumptions we are making about the temporal patterns of antiviral demand used in out Main Result:

 a)    For condition (ii) to imply the benefit of predispensing, we need that among the people who receive antivirals, the proportion of them who would die otherwise doesn’t change in time.

 b)    For conditions (iii) and (iv) to imply the benefit of predispensing, we need that among the people who receive antivirals, the proportion of them who would die otherwise doesn’t increase in time. 

 c)    For condition (i), no additional assumptions are needed.

Condition b) is more flexible than a) and can be interpreted as follows: consider persons infected during the epidemic. As time progresses, the proportion of them who will require antivirals may increase due to panic, etc. - suppose the increase is by a factor of \begin{equation*}X\end{equation*} . The people who would die without antivirals are the severe cases and the proportion of them who receives antivirals (given availability) is probably large - thus even if it increases it time, we assume that the increase is by a factor of at most \begin{equation*}X\end{equation*} . 

There are four conditions to consider: 

#### Condition (i): Supply exceeds demand even after predispensing \begin{equation*}T-q>D\end{equation*} and \begin{equation*}B>1\end{equation*}  

In this case all persons in the general population who demand antivirals would get them under the no predispensing scenario – thus predispensing would result in no loss of lives in that category compared to no predispensing. Since  \begin{equation*}B>1\end{equation*} , persons to whom antivirals were predispensed are more likely to survive under predispensing than through acquiring antivirals during the epidemic. Moreover no all among them who need antivirals would necessarily acquire them during the epidemic – thus predispensing is clearly beneficial. 

#### Condition (ii): In the absence of predispensing, demand exceeds supply, \begin{equation*}D>T\end{equation*}

To fix notation, let \begin{equation*}D'\geq D\end{equation*} be the demand for antivirals in the total population in the absence of predispensing. 

Among the \begin{equation*}q\end{equation*}  individuals in the high risk group, \begin{equation*}qRp_d\end{equation*} of them would die without antivirals. Out of those, \begin{equation*}qRBp_dp_s\end{equation*} will be saved by predispensed antivirals. Under no predispensing, not all of those \begin{equation*}qRp_d\end{equation*} people may demand antivirals, and among those who do, a fraction \begin{equation*}\frac{T}{D'}\leq \frac{T}{D}\end{equation*}  of them would get antivirals, so \begin{equation*}qRp_dp_s\frac{T}{D'}\end{equation*} lives would be saved (Table 2). 

TABLE 2: Numbers of lives saved in the general population and high risk group under no predispensing vs. predispensing, condition (ii) (demand exceeds supply)

**Table:** 

** **	**No predispensing**	**Predispensing**
General population	\begin{equation*}\frac{T}{D'}L\end{equation*}	\begin{equation*}\frac{T-q}{D}L\end{equation*}
High-risk group	at most \begin{equation*}\frac{T}{D'}qRp_dp_s\end{equation*}	\begin{equation*}qRBp_dp_s\end{equation*}

Turning now to the general population, if all the demand was met, the total number of lives saved without predispensing would be \begin{equation*}L\end{equation*}.  However, given scarcity, only   \begin{equation*}\frac{T}{D'}L\end{equation*} lives are saved (Table 2), because the remaining individuals do not receive antivirals.  If antivirals are predispensed, the remaining demand is \begin{equation*}D\end{equation*}  and the supply is \begin{equation*}T-q\end{equation*} . Thus a fraction \begin{equation*}\frac{T-q}{D}\end{equation*}  of  the demand in the general population is met, and the number of lives saved is thus \begin{equation*}\frac{T-q}{D}L\end{equation*} (Table 2).   

Using these figures (summarized in Table 2), there is a net gain in lives saved if the total number saved under predispensing exceeds that saved without predispensing, that is, if 

(A1)   $L\frac{T - q}{D} + qRBp_d p_s \geq L\frac{T}{D'} + qRp_d p_s \frac{T}{D'}$ . 

Rearranging and dividing by \begin{equation*}q>0\end{equation*} , we obtain the condition $L\left(\frac{1}{D} + \frac{T}{q}\left[ \frac{1}{D'} - \frac{1}{D}\right]\right)
            \leq Rp_d p_s B - Rp_d p_s \frac{T}{{D'}}$ .  Using the facts that \begin{equation*}D'\geq D\end{equation*}  and \begin{equation*}L \le p_s p_d \end{equation*} , a sufficient condition is then 

(A2) \begin{equation*}\frac{1}{D} \le R(B - T/D)\end{equation*}

which is condition (ii) so long as \begin{equation*}B>T/D\end{equation*}. 

#### **Condition (iii) $T - q \le D \le T$**

The argument is similar to the previous case. 

Among the high-risk group, without predispensing nearly all or all demand will be satisfied (there may be some competition if $D \le T \le D'$ ), so at most \begin{equation*}qRp_dp_s\end{equation*}  lives will be saved.  With predispensing, the situation will be as above. 

Among the general population, without predispensing, at most  \begin{equation*}L\end{equation*}  lives will be saved.   With predispensing, however, the situation will be as similar to Condition (ii), with equality replaced by inequality due to condition (b).  This is summarized in Table 3.  Repeating the comparison of equation (A1) for the entries in Table 3, we obtain Condition (iii). 

TABLE 3: Numbers of lives saved in the general population and high risk group under no predispensing vs. predispensing, Case 3 (supply exceeds demand before predispensing, but not after)

**Table:** 

** **	**No predispensing**	**Predispensing**
General population	at most \begin{equation*}L\end{equation*}	at least \begin{equation*}\frac{T-q}{D}L\end{equation*}
High-risk group	at most \begin{equation*}qRp_dp_s\end{equation*}	\begin{equation*}qRBp_dp_s\end{equation*}

#### **Condition (iv) no assumptions on demand.**

If $D \leq T$ , the result follows from Conditions (i) and (iii), since they use the same assumption b) as condition (iv). If $D>T$, the argument is similar to condition (iii), replacing $L$ by the number of individuals saved by the first $T$ courses of antivirals in the general population. 

### B.  Formal description of the parameters \begin{equation*}p_s\end{equation*} , \begin{equation*}R\end{equation*}  and \begin{equation*}B\end{equation*} .   

Suppose that for every individual who is either a member of the high risk group \begin{equation*}G=h\end{equation*}  or the total population \begin{equation*}G=w\end{equation*} , we can define an outcome \begin{equation*}O(T)\end{equation*}  that depends on the (possibly counterfactual) treatment \begin{equation*}T\end{equation*} received, where either \begin{equation*}O=d\end{equation*}  (died) or \begin{equation*}O=s\end{equation*}  (survived) and either  \begin{equation*}T=U\end{equation*} (untreated), or \begin{equation*}T=S\end{equation*}  (treated from the stockpile), or \begin{equation*}T=P\end{equation*} (treated from a predispensed course, and that course has not been previously wasted).  Then

\begin{equation*}\[ \begin{array}{l} p_D = \Pr [O(U) = d|G = w] \\ p_S = \Pr [O(S) = s|O(U) = d] =
            \Pr [O(S) = s|O(U) = d,G = w] = \Pr [O(S) = s|O(U) = d,G = h] \end{array} \]\end{equation*}

\begin{equation*}\[ \begin{array}{l} R = \frac{{\Pr [O(U) = d|G = h]}}{{\Pr [O(U) = d|G = w]}} \\ B
            = \frac{{\Pr [O(P) = s|O(U) = d,G = h]}}{{\Pr [O(S) = s|O(U) = d,G = h]}} \\ \end{array}
            \]\end{equation*}

We are interested in the probability that treatment (nonpredispensed) will save a life in the total population, which is

\begin{equation*}\[ p_d p_s = \Pr [O(U) = d,O(S) = s|G = w] \]\end{equation*},

in the probability that treatment without predispensing will save a life in the high-risk group, which is

\begin{equation*}\[ \begin{array}{l} p_D p_S R = \Pr [O(U) = d|G = h]\frac{{\Pr [O(U) = d|G =
            h]}}{{\Pr [O(U) = d|G = w]}}\Pr [O(S) = s|O(U) = d,G = h] \\ = \Pr [O(S) = s,O(U) = d|G
            = h] \\ \end{array} \]\end{equation*}

  

and in the probability that a high risk person who received a predispensed course would thereby have his life saved, which is

\begin{equation*}\[ p_D p_S RB = \Pr [O(T) = s,O(U) = d|G = h] \]\end{equation*}

### **C. When does the benefit increase with the number of courses predispensed?**

While predispensing to high risk groups is beneficial compared to no predispensing, if condition (iv) holds, it is not true in general that the benefit of predispensing increases with the quantity of antivirals predispensed. Consider for example one high risk group whose size equals (or surpasses) the antiviral supply.  As we keep on predispensing, coverage level for the rest of the population goes to 0  while the relative risk of dying in the high risk group stays uniformly bounded. Thus at some point equation (iv) will be violated (with the new coverage level and the relative risk), and in fact it is not advisable to predispense the whole supply to the group under no a-priori assumptions on the demand  \begin{equation*}D\end{equation*} . However the have the following simple criterion:

BENEFIT INCREASES WITH THE AMOUNT PREDISPENSED: 

Suppose the relative risk of dying  $R \ge 1$ , and assumption b) in Appendix A holds. The benefit of predispensing is guaranteed to increase with quantity of antivirals predispensed as long as the total quantity \begin{equation*}q\end{equation*}of antivirals predispensed obeys 

(C1)$\frac{{T - q}}{{1 - q}} \ge \frac{1}{{R(B - 1)}}$  

To show this, let the quantity \begin{equation*}q\end{equation*}  of antivirals predispensed obey the inequality (C1). To show that the benefit of predispensing was increasing, take any intermediate quantity \begin{equation*}q_I\leq q\end{equation*} . We need the show that equation (C1) was still true after predispensing \begin{equation*}q_I\end{equation*}  courses of antivirals. Note that after this predispensing, the coverage level and the relative risks have changed. The coverage level became 

(C2) \begin{equation*}\[ T_I = \frac{{T - q_I }}{{1 - q_I }} \ge \frac{{T - q}}{{1 - q}} \ge
            \frac{1}{{R(B - 1)}} \]\end{equation*}   

Also, as a result of predispensing, we've removed some individuals with the risk of dying higher than the one for the general population. Thus the probability of dying for an average individual left after predispensing is lower than the one before predispensing; hence the relative risks of the high risk groups have only increased. Since equation (C2) for the new coverage level holds with original relative risks, condition (iv) in our Main Result holds with the new coverage levels and relative risks (after predispensing \begin{equation*}q_I\end{equation*} courses of antivirals). This implies that predispensing \begin{equation*}q-q_I\end{equation*}  courses after predispensing the initial \begin{equation*}q_I\end{equation*}  courses would be beneficial. 

## References

[ref-3070768605] Aoki FY, Macleod MD, Paggiaro P, Carewicz O, El Sawy A, Wat C, Griffiths M, Waalberg E, Ward P, IMPACT Study Group: Early administration of oral oseltamivir increases the benefits of influenza treatment. J Antimicrob. Chemother. 2003; 51(1) 123-9 10.1093/jac/dkg007 12493796

[ref-2509778060] Burch J, Corbett M, Stock C, Nicholson K, Elliot AJ, Duffy S, Westwood M, Palmer S, Stewart L: Prescription of anti-influenza drugs for healthy adults: a systematic review and meta-analysis. Lancet Infect Dis. 2009 Aug 7. [Epub ahead of print] 19665930 10.1016/S1473-3099(09)70199-9

[ref-2593680428] McClellan K, Perry CM, Oseltamivir: a review of its use in influenza. Drugs. 2001;61(2):263-83. 10.2165/00003495-200161020-00011 11270942

[ref-2321005289] Kaiser L, Wat C, Mills T, Mahoney P, Ward P, Hayden F. Impact of oseltamivir treatment on influenza-related lower respiratory tract complications and hospitalizations. Arch Intern Med. 2003 Jul 28;163(14):1667-72. 10.1001/archinte.163.14.1667 12885681

[ref-1556464731] Jain S, Kamimoto L, Bramley AM, Schmitz AM, Benoit SR, Louie J, et al. Hospitalized Patients with 2009 H1N1 Influenza in the United States, April-June 2009. N Engl J Med 2009 Oct 8. 10.1056/NEJMoa0906695 19815859

[ref-3562892496] Janice K. Louie; Meileen Acosta; Kathleen Winter; et al. Factors associated with death or hospitalization due to pandemic 2009 influenza A(H1N1) infection in California. JAMA. 2009;302(17):1896-1902 (doi:10.1001/jama.2009.1583) 10.1001/jama.2009.1583 19887665

[ref-316639952] CNN. Oct. 13, 2009 Study: Underlying conditions playing less of role in H1N1 http://www.cnn.com/2009/HEALTH/10/13/h1n1.preexisting.conditions/index.html

[ref-2436736239] Jamieson DJ, Honein MA, Rasmussen SA, Williams JL, Swerdlow DL, Biggerstaff MS, et al. H1N1 2009 influenza virus infection during pregnancy in the USA. Lancet. 2009 Aug 8;374(9688):451-8. 10.1016/S0140-6736(09)61304-0 19643469

[ref-1590500135] Ministerio de Salud, Chile. Influenza pandemica (H1N1) 2009. REPORTE 21 DE OCTUBRE DE http://www.redsalud.gov.cl/minsalaudios/reporte22octubre.pdf

[ref-1504385554] Perez-Padilla R, de la Rosa-Zamboni D, Ponce de Leon S, Hernandez M, Quinones-Falconi F, Bautista E, et al. Pneumonia and respiratory failure from swine-origin influenza A (H1N1) in Mexico. N Engl J Med2009 Aug 13;361(7):680-9. 10.1056/NEJMoa0904252 19564631

[ref-1239612372] Rello J, Rodriguez A, Ibanez P, Socias L, Cebrian J, Marques A, et al. Intensive care adult patients with severe respiratory failure caused by Influenza A (H1N1)v in Spain. Crit Care2009 Sep 11;13(5):R148 10.1186/cc8044 PMC2784367 19747383

[ref-166664201] H1N1 leading to increased flu rates, more pediatric deaths. http://www.rwjf.org/publichealth/digest.jsp?id=23828

[ref-3139123044] CDC. Updated Interim Recommendations for the Use of Antiviral Medications in the Treatment and Prevention of Influenza for the 2009-2010 Season. http://www.cdc.gov/h1n1flu/recommendations.htm

[ref-3427677528] Public Health Agency of Canada. Use of antivirals to treat H1N1 flu virus. http://www.phac-aspc.gc.ca/alert-alerte/h1n1/antiviral-antiviraux05-01-eng.php

[ref-776221430] Ministère chargée de la santé. Prise en charge d’un cas de grippe A( H1N1) chez un adulte ou un enfant de plus de 40 kg. Grippe, formation, information, communication. Fiche mémo; 2009; http://www.sante-sports.gouv.fr/IMG//pdf/Prise_en_charge_d_un_cas_de_grippe_A_H1N1_chez_un_adulte_ou_un_enfant_de_plus_de_40_kg.pdf

[ref-2030528610] Department of Health and Ageing, Australia. Antiviral medication use – treatment. http://www.healthemergency.gov.au/internet/healthemergency/publishing.nsf/Content/clinical-antiviral

[ref-2242979749] National Health Coordination Center, New Zealand. Guidance on the diagnosis and management of Pandemic (H1N1) 2009 in the Pandemic ‘Management’ phase, Version 3. http://www.moh.govt.nz/moh.nsf/pagesmh/9171/$File/diagnosis-and-mgmt-guidance-for-mgmt-phase-v3-30july09.doc

[ref-1110531368] MIDAS High Risk Segmentation Group. (D. Wagener, private communication, 2009).

[ref-3267758521] Ogden CL, Carroll MD, Curtin LR, McDowell MA, Tabak CJ, Flegal KM. Prevalence of overweight and obesity in the United States, 1999-2004. JAMA. 2006; 295(13):1549-1555. 10.1001/jama.295.13.1549 16595758

[ref-2562081569] CDC. A weekly influenza surveillance report prepared by the influenza division. http://www.cdc.gov/flu/weekly/

[ref-1047776214] Keren R, Zaoutis TE, Bridges CB, et al. Neurological and neuromuscular disease as a risk factor for respiratory failure in children hospitalized with influenza infection. JAMA 2005;294:2188-2194 10.1001/jama.294.17.2188 16264160

[ref-374826205] Toovey S, Rayner C, Prinssen E, Chu T, Donner B, Thakrar B, Dutkowski R, Hoffmann G, Breidenbach A, Lindemann L, Carey E, Boak L, Gieschke R, Sacks S, Solsky J, Small I, Reddy D. Assessment of neuropsychiatric adverse events in influenza patients treated with oseltamivir: a comprehensive review. Drug Saf. 2008;31(12):1097-114. doi: 10.2165/0002018-200831120-00006. Review. 19026027 10.2165/0002018-200831120-00006

[ref-2583398228] Lipsitch M, Samore MH. Antimicrobial use and antimicrobial resistance: a population perspective. Emerg Infect Dis. 2002 Apr;8(4):347-54. 10.3201/eid0804.010312 PMC2730242 11971765

[ref-779459754] Weekly pandemic flu media update. Health Protection Agency. 22 October 2009 http://www.hpa.org.uk/web/HPAwebFile/HPAweb_C/1254510639308

[ref-3773165249] Lipsitch M, Cohen T, Murray M, Levin BR (2007) Antiviral Resistance and the Control of Pandemic Influenza. PLoS Med 4(1): e15. http://www.plosmedicine.org/article/info:doi/10.1371/journal.pmed.0040015 10.1371/journal.pmed.0040015 PMC1779817 17253900

[ref-1040828799] Wu JT, Leung GM, Lipsitch M, Cooper BS, Riley S (2009) Hedging against Antiviral Resistance during the Next Influenza Pandemic Using Small Stockpiles of an Alternative Chemotherapy. PLoS Med 6(5): e1000085. http://www.plosmedicine.org/article/info:doi%2F10.1371%2Fjournal.pmed.1000085 10.1371/journal.pmed.1000085 PMC2680070 19440354

[ref-3446914576] Presanis AM, Lipsitch M, DeAngelis D et. al. The severity of pandemic H1N1 influenza in the United States, April – July 2009 http://knol.google.com/k/anne-m-presanis/the-severity-of-pandemic-h1n1-influenza/agr0htar1u6r/16# 10.1371/currents.RRN1042 PMC2762775 20029614

[ref-3342172176] Washington Post. Oct 2, 2009. CDC Reports 28 Flu Deaths Among Pregnant Women. http://www.washingtonpost.com/wp-dyn/content/article/2009/10/01/AR2009100104308.html

[ref-3911190679] Swine flu poses risks to kids with neurological conditions. Medline Plus, Sep. 3, 2009 http://www.nlm.nih.gov/medlineplus/news/fullstory_88952.html

[ref-1200866011] E. Goldstein, M. Lipsitch. Antiviral usage for H1N1 treatment: pros, cons, and an argument for broader prescribing guidelines in the United States. PLOS Currents Influenza. http://knol.google.com/k/edward-goldstein/antiviral-usage-for-h1n1-treatment-pros/1dfgb8wq76hv/2# 10.1371/currents.RRN1122 PMC2770576 20029660

[ref-3893845834] Goldstein E, Cowling B, O’Hagan J, Danon L, Fang V, Hagy A, Miller JC, Reshef D, Robins J, Biedrzycki P, Lipsitch M. Oseltamivir for treatment and prevention of pandemic influenza A/H1N1 virus infection in households, Milwaukee, 2009, Submitted. http://www.plosmedicine.org/article/info:doi%2F10.1371%2Fjournal.pmed.1000085 10.1186/1471-2334-10-211 PMC2919545 20642862

